# Apoptosis in human tumours

**DOI:** 10.1038/sj.bjc.6600312

**Published:** 2002-05-03

**Authors:** C A Rubio

**Affiliations:** Gastrointestinal and Liver Pathology, Research Laboratory, Karolinska Institute and Hospital, 171 76 Stockholm, Sweden

## Abstract

*British Journal of Cancer* (2002) **86**, 1661–1661 DOI: 10.1038/sj/bjc/6600312 www.bjcancer.com

© 2002 Cancer Research UK

## Sir

We read with interest the article by Hadjiloucas *et al* (2001) on apoptosis in human breast tissue using an antibody against caspase 3. They recognise that ‘many methods are available for the measurement of apoptosis but the ‘gold standard’ is to identify apoptotic cells by their morphological features using microscopy’ … Moreover, ‘the TUNEL method fails to distinguish apoptosis from necrosis, and only detects lat stages of the process, and furthermore can produce artefactual positive results where nuclei are cut during the sectioning procedure’.

The authors found, on the other hand, that the use of caspase 3 immunostain is ‘a robust method for assessing in biopsies from breast epithelia’. For many years we have investigated apoptosis in colorectal neoplasms, with the TUNEL method, as well as with immunostains such as p53, CD3, MCHII, TIA1 cell antigens and Fas methods (Rubio *et al*, 1996; Rubio, 1997). In colorectal neoplasms, particularly in adenomas, the results indicated that the apoptotic nuclear granules (known as Leuchtenberger or Councilman bodies) derive not from the epithelial cells but from the intraepithelial lymphocytes which often infiltrated the neoplastic cells. The TUNEL method labelled some apparently well preserved neoplastic cells. Proliferation studies with an antibody to Ki67 (MIB1) showed that the majority of the neoplastic epithelial cells were actively synthesising DNA (thus conceivably viable, apparently not ‘planning’ for an imminent programmed cell death).

On the other hand, CD3, MCHII and TIA1 cell antigens were positive in intraepithelial lymphocytes (IELs) from adenomas, incipient carcinomas and overt adenocarcinomas (Rubio *et al*, 1999). In the normal colorectal mucosa and in metaplastic polyps, CD3 was positive but MCHII and TIA1 were not expressed. Whereas the Fas cell antigen was expressed in the normal mucosa and in metaplastic polyps, it was downregulated in neoplastic lesions. The upregulation of CD3, MCH class II molecules, and TIA1 in IELs of neoplastic colorectal lesions suggested that those lymphocytes were activated and cytotoxic. However, their Fas ligand was unable to induce apoptosis on epithelial neoplastic cells because the Fas molecule of neoplastic cells was downregulated. In contrast, the Fas ligand of the neoplastic cells was able to induce the apoptosis of IELs because the Fas molecule of IELs remained intact. A similar phenomenon seems to take place in experimentally-induced colonic neoplasias in rats (Rubio, 1998). Thus, the possibility that lymphocytes may have contributed to the bulk of apoptotic granules in breast tumours (alas! also infiltrated by lymphocytes) cannot be disregarded. Leuchtenberger or Councilman bodies are just bodies; they do not disclose if they are of epithelial or of lymphatic origin. In this respect, it may be of interest to mention that Liang *et al* (2001) recently found that human breast MCF-7 cancer cells do not express caspase 3.

Interestingly, Hadjiloucas *et al* (2001) concluded that ‘the appearance of the active form of caspase 3 in the cytoplasm of the cells undergoing apoptosis is an early event and precedes the development of the classical morphological features of apoptosis’.

A similar argument was used many years ago upon the launching of the TUNEL method.
